# A call for switching to a 1-dose 9vHPV national vaccination program in Ethiopia

**DOI:** 10.3389/fpubh.2023.1211894

**Published:** 2023-08-07

**Authors:** Tesfaye Gelanew, Liya Wondwossen, Adane Mihret, Andargachew Mulu

**Affiliations:** ^1^Armauer Hansen Research Institute, Addis Ababa, Ethiopia; ^2^Federal Ministry of Health, Addis Ababa, Ethiopia

**Keywords:** cervical cancer, HPV, genotype, girls, HIV, vaccine, dose, Ethiopia

## Background

Cervical cancer (CC), caused by a group of human papillomaviruses (HPV), is primarily a sexually transmitted disease. In Ethiopia, CC is the second most common cancer with estimated annual new cases and deaths of 7,450 and 5,300, respectively (1). CC and other high-risk (HR), probable-risk (PR), and low-risk (LR) carcinogenic genotypes-associated diseases are vaccine-preventable. Currently, six commercial prophylactic HPV vaccines are available: three bivalent (HPV 16 and 18) vaccines (2vHPV), two quadrivalent (HPV 6, 11, 16, and 18) vaccines (4vHPV), and a nonavalent (HPV 6, 11, 16, 18, 31, 33, 45, 52, and 58) vaccine (9vHPV) (2). All these vaccines were originally licensed as a three-dose (3-dose) schedule over six months intervals, but a two-dose (2-dose) schedule was approved in girls younger than 15 years in 2016 after post-license efficacy evidence became available (3, 4). Subsequently, Ethiopia launched the HPV vaccination program with 2-dose Gardasil^®^4 (4vHPV vaccine) in December 2018 with the support of Gavi, the Global Vaccine Alliance.

Like many Gavi- eligible countries, Ethiopia had to choose a single-age cohort primarily targeting schoolgirls aged 14 years old because of the global vaccine supply constraint. The supply constraint is projected to continue for several years with many Gavi-eligible countries planning the introduction of HPV national immunization programs (5). Importantly, WHO set a goal of achieving CC elimination by 2030 worldwide. A dose-reduction vaccination approach is urgently needed if the WHO goal of CC elimination by 2030 is to be met. High-quality large-scale observational studies in Costa Rica and India demonstrated that a one-dose (1-dose) HPV vaccine regimen induces similar long-term protection to that of multidose (3, 4). On 7th April 2022, based on these efficacy data, the WHO's Strategic Advisory Group of Experts on Immunization (SAGE) recommended that each country could decide to implement alternative, off-label regimens, including a 2-dose schedule in all age groups or a 1-dose schedule for individuals aged 9–20 years (6). Before the implementation of a 1-dose HPV vaccine regime, however, the following key policy questions should be answered based on the currently available evidence in the Ethiopian context: (i) which 1-dose vaccine type regime (1-dose 4vHPV versus 1-dose 9vHPV) is optimal; (ii) does Ethiopia need locally generated evidence to make a policy switch to a 1-dose 9vHPV vaccine regime; (iii) will 1-dose 9vHPV vaccine be sufficient to offer protection to HIV-infected girls; and (iv) is the HPV vaccine uptake optimal? In this opinion paper, we provide our insights related to these key policy questions.

## Single-dose 9vHPV vaccine will offer protection to more commonly circulating HR-HPV genotypes

According to the recent systematic review, HPV 16 is the most dominant HPV genotype among HPV-positive women in Ethiopia, accounting for over 37% of HR-HPV infections while HPV18 is responsible for 4.4% of infections (Table 1) (7) with a combined HPV16/18 infection prevalence of 54%. The seven common non-HPV16/18 HR-HPV genotypes detected in Ethiopia were [HPV-31 (3.8%), 33 (1.7%), 35 (4.8%), 39 (1.9%), 45 (3.5%), 52 (6.8%), 58 (3.1%), and 68 (2.8%)] (7). The data implies that 46% of women's infections with HR-HPV genotypes in Ethiopia are related to non-HPV 16/18 genotypes. A very recent study found that HPV16 (31.8%), 31 (19.1%), 52 (11.8%), 58(10.9%), and 35 (10%) were the most frequently detected HR-HPV genotypes while HPV18 was detected in only 2.7% of HPV-positive women (Table 1) (8). These data altogether suggest that vaccinating girls with the 4vHPV vaccine would protect only 54 % of infections with HR-HPV genotypes, leaving 46% of vaccines vulnerable to infections with non-4vHPV vaccine HR-HPV genotypes. By contrast, the 9vHPV vaccine is highly efficacious in offering protection to five additional HR-HPV genotypes (9). Thus, if Ethiopia switches to the 9vHPV vaccine, over 90% of vaccinated women will likely be protected from the 9vHPV vaccine HR-HPV genotypes-related persistent infections and diseases (Table 1).

**Table 1:** Relative distribution of 9vHPV vaccine HPV genotypes in women with and without abnormal cervical dysplasia in Ethiopia and potential protection offered by 9vHPV and 4vHPV vaccination.

**9vHPV vaccine HPV genotype**	**Proportion (%) of 9vHPV vaccine genotypes [Derbie et al. (7)^a^]**	Potential protection offered by 9vHPV^*^vaccine	**Potential protection offered by 4vHPV^§^vaccine**	**Proportion (%) of 9vHPV vaccine genotypes [Seyoum et al. (8)^b^]**	**Potential protection offered by 9vHPV vaccine**	Potential protection offered by 4vHPV^*^vaccine
**HR-HPV**						
HPV 16	37.0	+	+	31.8	+	+
HPV 18	4.4	+	+	2.7	+	+
HPV 31	3.8	+	–	19.1	+	–
HPV 33	1.7	+	–	2.7	+	–
HPV 45	3.5	+	–	6.4	+	–
HPV 52	6.8	+	–	11.8	+	–
HPV 58	3.1	+	–	10.9	+	–
**LR-HPV**						
HPV 6	2.0	+	+	UK	+	+
HPV 11	2.7	+	+	UK	+	+

a systematic review;

b a hospital-based cross-sectional study;

* proposed Gardasil 9vHPV vaccine;

§ currently in-use Gardasil 4v HPV vaccine; + offers protection; – offers little or no protection; UK, unknown; HR-HPV, high-risk oncogenic HPV genotypes; low-risk HPV genotypes which can cause anogenital warts.

## Evidence for the efficacy of 1-dose 9vHPV vaccine in east Africa in HIV-negative cohorts

Given obtaining efficacy data based on virological or cervical dysplasia (disease) endpoints will take several years when vaccination given to girls before first sexual debut, the efficacy of the 1-dose 9vHPV vaccine can be assessed through immunobridging trials (10). An immunobridging trial is a non-inferiority comparison of geometric mean concentrations (GMCs) of anti-HPV antibodies for specific HPV genotypes in a new population group with those in a population group in whom efficacy had been established in randomized clinical trial (RCT) with virological or disease endpoints. It assumes if the GMCs of antibodies between two groups are comparable, the efficacy between the two groups is comparable too. Accordingly, immunobridging RCTs on a 1-dose 9vHPV vaccine schedule have been conducted in sub-Saharan African countries where the 90% of the global CC burden occurs and dose reduction is a critical strategy for increasing HPV vaccine coverage (10). Recent randomized muti-center, double-blind, controlled trials with clinical endpoints at 35 months in Tanzania (11) and at 18 months in Kenya (12) indicate 96.0% and 91.4% efficacies, respectively in the HPV 16/18/31/33/45/52/58, substantiating the evidence obtained from immunobridging RCT in Tanzania. These results from RCTs conducted in Tanzania and Kenya, where like Ethiopia their populations have similar additional comorbidities (e.g., HIV, parasites, and malnutrition) that may compromise the quality and durability of vaccine-induced immune responses, proved the efficacy of 1-dose 9vHPV vaccine in preventing CC associated with infections with HPV-16, 18, 31, 33, 45, 52, and 58. Based on these established efficacy results along with the diversity of HR-HPV genotypes distribution data in Ethiopia, we are advocating a policy switch to a 1-dose 9vHPV vaccine schedule. Nevertheless, if the Ministry of Health needs prior local evidence to make the in-demand decision, conducting an immunobridging RCT study that aims to compare antibody GMC specific to HPV genotypes with the Tanzanian 1-dose 9vHPV vaccine immunobridging cohort (10) can be considered instead of waiting for several years until local efficacy data based on virological or disease endpoints will be generated.

## Will 1-dose 9vHPV vaccine be sufficient for HIV-infected girls?

In sub-Saharan Africa, including Ethiopia, the burden of HIV infection is also high. In 2023 alone, >620,000 new HIV infections are estimated to occur in Ethiopia with women being disproportionately infected (13). Approximately 1 in 5 CCs occur in women living with HIV (WLWH). Additionally, WLWH are more likely to be infected with more HR-HPV genotypes not covered by the 4vHPV vaccine (14). Considering this, WLWH will more likely benefit from the introduction of the 9vHPV vaccine. Despite accumulated evidence on immunogenicity and tolerability of 3-dose of both 4vHPV and 9vHPV vaccines in HIV-infected children and women (15), however, there is no RCT regarding the efficacy of 1-dose 9vHPV vaccine in HIV-positive individuals so far. An immunobridging study would be the fastest strategy to get approval for the use of a 1-dose 9vHPV vaccine in WLWH. Until results from immunobridging studies are publicly available, we dither advocating the use of 1-dose 9vHPV in HIV-infected girls. Instead, we recommend at least 2-dose 9vHPV vaccination over the ongoing 2-dose 4vHPV given the 9vHPV vaccine's potential to offer protection to more non-4vHPV vaccine HR-HPV genotypes (9).

## Economic benefits of 1-dose 9vHPV vaccine

Besides the additional public health benefits, a mathematical model-based study suggests the cost-effectiveness of the 9vHPV vaccine in Ethiopia compared to the current 2-dose 4vHPV vaccination program (16). Another modeling analysis showed similar economic beneficial impact of a 1-dose of 9vHPV vaccination schedule in Tanzania (17), where like Ethiopia, high diversity of HR HPV genotypes has been detected in women with abnormal cervical lesions. The minimum price per dose for Gardasil 4vHPV and 9vHPV vaccines are $4.50 and $5.18, respectively (16). In Ethiopia, the estimated number of girls of age 14 years who are eligible to take the current 2-dose 4vHPV vaccine in 2023 alone is above 1.5 million (13). If Ethiopia adopts a 1-dose 9vHPV vaccine as its national HPV immunization schedule, it will save a minimum of $5.73 million per year and a total of $40.11 million by end of 2030, assuming that the vaccine uptake and coverage is 100% and the number of HPV vaccine eligible girls remain the same. In addition, it will save the costs required for second dose injection needle and delivery, including personnel, transportation and storage costs. And this saved money can be stretched to support vaccine delivery or building other healthcare services.

## Vaccine uptake and barriers to reaching the optimal level

Despite the excellent safety profile of all HPV vaccines, its uptake in Ethiopia is suboptimal as low as 44.4% (18). In addition, a low disparity between uptake of 1-dose and 2-dose was observed (19), suggesting that switching to a 1-dose schedule alone may not necessarily increase HPV vaccination coverage to an optimal level. The main barriers to vaccine uptake were concerns about side effects (primarily fear of injection and pain at the site of injection), a misconception that the vaccine is given as anti-fertility, and a lack of adequate information on the benefit of the HPV vaccine before the vaccination schedule and lack of regular vaccination program (18, 19). However, barriers related to vaccine side effects can be circumvented by dose reduction as girls will prefer a 1-dose over 2-dose schedule to avoid pain caused by the second shot. Future country-wide study that involves all stakeholders is urgently needed to identify the main barriers to single-dose vaccine uptake and mitigation strategies.

## Conclusions and recommendations

Until the global vaccine shortage is circumvented and becomes affordable to low and middle-income countries (LMICs), including Ethiopia, we suggest switching to a 1-dose 9vHPV vaccination program to accelerate the elimination of CC in Ethiopia by 2030. For HIV-infected girls, however, we recommend the use of at least a 2-dose 9vHPV vaccination schedule until evidence regarding the efficacy of 1-dose 9vHPV vaccination in WLWH are available. Switching to a 1-dose vaccination schedule can also partially circumvent the current suboptimal HPV vaccine uptake in Ethiopia.

Despite its health benefits, the 9vHPV vaccine is not yet included in the Gavi support list for low-income countries (LICs). Thus, high CC burden LICs, including Ethiopia with a high prevalence of non-4vHPV vaccine HR-HPV genotypes should request Gavi to consider the 9vHPV vaccine in its support list or seek other global international support. If Gavi requires local evidence to support switching to a 1-dose 9vHPV vaccine schedule, Ethiopia may consider conducting a pilot immunobridging RCT instead of clinical efficacy data.

## Author contributions

Conceptualization: TG and AMu. Writing of the initial draft of the manuscript: TG. Editing and reviewing the last draft: TG, LW, AMi, and AMu.
